# Radiographic localization of supernumerary teeth: a narrative review

**DOI:** 10.3389/fdmed.2025.1495025

**Published:** 2025-02-10

**Authors:** Sreekanth Kumar Mallineni, Robert Prashanth Anthonappa, Jayakumar Jayaraman, Nigel Martyn King

**Affiliations:** ^1^ Pediatric Dentistry, Dr. Sulaiman Al Habib Medical Group, Ar Rayyan, Riyadh, Makkah Province, Saudi Arabia; ^2^ School of Dentistry, The University of Western Australia, Nedlands, WA, Australia; ^3^ Department of Pediatric Dentistry, Virginia Commonwealth University School of Dentistry, Richmond, VA, United States

**Keywords:** localization, radiographs, impacted teeth, supernumerary teeth, CBCT

## Abstract

**Objectives:**

To conduct a narrative review of the published literature on the localization techniques to identify the best technique for the localization of supernumerary teeth.

**Methods:**

An extensive search of literature published in English between January 1966 and May 2024 was conducted using the “Medline via PubMed” and “Cochrane database” databases. The keywords included in the search were “localization” “radiographs”, and “impacted teeth”, or “un-erupted teeth”, or “supernumerary teeth” or “supernumerary tooth”. The citation lists from the included articles were examined to identify additional reports and a hand search was also conducted. Kappa statistics were used for intra-examiner reliability.

**Results:**

The initial search yielded 4,864 citations, subsequently examined and supplemented by a hand search to find additional studies. Commonly used techniques for the localization of supernumerary teeth are the horizontal tube shift technique, vertical tube shift technique, vertex occlusal technique, and cone-beam computerized tomography.

**Conclusion:**

The most commonly used localization techniques for supernumerary teeth are horizontal tube shift, vertex occlusal, vertical tube shift, and cone-beam computerized tomography. Conventional radiographs only provide two-dimensional views of 3-dimensional structures. Three-dimensional imaging alone can provide accurate data on impacted supernumerary teeth but, the need for this film has to be justified because of the higher radiation exposure.

## 1 Introduction

Supernumerary teeth are defined as “any tooth or odontogenic structure that is formed from a tooth germ in excess of the usual number for any given region of the dental arch” (1). They may be single or multiple, unilateral or bilateral in distribution, and can occur in both dental arches, and either in the primary mixed or permanent dentitions. The mesiodens is the most commonly occurring supernumerary tooth followed by mandibular premolars, which are the supernumerary teeth of the supplemental type (2–4). Males are more commonly affected than females, at a ratio of 2:1 (5, 6). Several hypotheses have been proposed to explain the occurrence of supernumerary teeth, but their etiology remains unclear (1, 7). According to one systematic review, the prevalence of supernumerary teeth ranges from 1.5% to 3% in the general population with a predilection to the mongoloid racial group (8). Early identification and appropriate management are critical to either limit or prevent the consequences of supernumerary teeth, which range from crowding to cyst formation. Clinical diagnosis is the primary and most important aid in the diagnosis of an impacted tooth (9, 10). Clinical localization includes visual inspection and palpation, while radiographic localization is based on different combinations of radiographs (11). Localization of supernumerary teeth plays a major role in diagnosis and treatment planning, especially if surgical intervention is required (12). Although early intervention can potentially prevent later complications, several authors have been cited as having anecdotally suggested that this approach is harmful due to the possible risk of damage to the developing tooth germs (1, 2). The location of supernumerary teeth can be confirmed by using a variety of imaging techniques. The interpretation principle “SLOB” (Same Lingual Opposite Buccal) rule is the most commonly used one when applying the concept of parallax (13). Localization of an un-erupted tooth is based on a combination of clinical and radiographic assessment (14). The more exact the localization of supernumerary teeth, potentially the less invasive the surgical procedure; therefore, the purpose of this paper was to conduct a narrative review of the available literature and to identify the best technique for the localization of supernumerary teeth.

## 2 Materials and methods

An extensive search of literature published in English between January 1966 and May 2024 was conducted using the “Medline via PubMed” and “Cochrane database” databases. The keywords included in the search were “localization” and “radiographs”, and “impacted teeth”, “un-erupted teeth”, or “supernumerary teeth” or “supernumerary tooth”. The citation lists from the included references were subsequently examined, in addition hand searching was performed in an attempt to identify additional papers. Kappa statistics were used for intra-examiner reliability.

## 3 Results

The initial search yielded 4,873 citations from PubMed Medline and 27 from Cochrane database, which were subsequently examined and supplemented by a hand search to find additional studies. Eighteen articles were available for final analysis on the localization of supernumerary teeth five related to horizontal tube shift technique (HTST) (5), six each related to vertical tube shift technique (VTST) and cone beam computerized tomography (CBCT) (6), and one related to computerized tomography (CT). No literature was evident for panoramic radiographs alone and magnetic resonance imaging (MRI) for the localization of supernumerary teeth. The most commonly used techniques for the localization of supernumerary teeth are Clark's technique (13), vertex occlusal (15), and Keur's technique (16), all of which involve the use of conventional radiographs (Table 1). Three-dimensional radiographs for localization and dimensional evaluation include CBCT, CT, MRI, Spiral computerized tomography (SCT), Scanora, and dental magnetic resonance imaging (dMRI), of which CBCT is most often used for the localization of un-erupted impacted/supernumerary teeth in the anterior region of the maxilla (Table 1). Only one reviewer was involved in the literature search and Kappa statistics showed good intra-examiner reliability (K = 0.89). Various localization techniques have been described in the literature (9, 11, 13, 15–42). The most commonly used ones for the localization of supernumerary teeth (13, 15–17), and all the other techniques used for localization of impacted teeth have been summarized in Table 2. The most frequently used localization techniques are described in detail in the text.

**Table 1:** Commonly used methods for the localization of supernumerary teeth.

Authors	Year	Method	Radiographs
Clark (13)	1910	Horizontal technique	3 PA
Hitchin (15)	1951	Vertex occlusal	AO
Keur (16)	1986	Vertical tube shift	PAN and AO
Mozzo et al. (17)	1998	CBCT	

PA, periapical radiograph; AO, anterior occlusal radiograph; PAN, panoramic radiograph; CBCT, cone beam computerised tomography.

**Table 2:** Different combinations of radiographs used for the localization of impacted teeth.

Authors	Year	Methods	Radiographs
Mackenzie Davidson (18)	1898	Stereoscopy	2 PA or 2 AO
Bosworth (19)	1934	Multiple exposures	1PA
Donovan (20)	1952	Occlusal radiography	AO
Richards (21)	1952	Buccal object rule	2PA
Broadway and Gould (22)	1960	Ballard suggestion for localization	LC and PAC
Seward (23)	1963	Radiology in general practice	Apex PA, VO, PA skull, ROL of jaws, and lateral exposure of sinus
Rayne (9)	1969	Localization of canine	PA and AO
Wraith (24)	1969	Radiographic assessment canines	PA skull, LC and 2 PA
Turk and Katzenell (25)	1970	Panorex	PAN
Hounsfield (26)	1973	Computerized tomography	
Ostrofsky (27)	1976	Magnification technique	PAN
Beeching (28)	1981	Parallax with Panorex	PAN, VO of upper jaw or AO of the lower jaw
Coupland (29)	1984	LC skull and PAN	LC and PAN
Keur (16)	1986	Keur technique	2AO
Ericson and Kuroll (30)	1986	Polytomography	
Southall and Gravely (31)	1989	Vertical parallax radiology	OO and PAN
Miller et al. (32)	1990	Cross-sectional tomography	
Jensen (33)	1990	Free-focus radiography	PAN
Tammisalo et al. (34)	1992	Scanora	
Ong (35)	1994	Alternative to VO	AO
Felice and Lombardi (36)	1995	Water's view	Water's view and PAN
Gray et al. (37)	1996	MRI	
Preda et al. (38)	1997	Spiral CT	
Jacobs (11)	1999	Right angle technique	PAN and AO
Jacobs (39)	2000	Cross- sectional occlusal radiography	AO and PAN
Kim et al. (40)	2003	SLUOBD method	PAN and PA
Tony and Alfred (41)	2010	Tangential radiography	PAN and AO
Tymofiyeva et al. (42)	2010	dMRI	

PA, periapical radiograph; AO, anterior occlusal; VO, vertex occlusal; OO, oblique occlusal; LC, lateral cephelogram; PA skull, posteroanterior view of skull; PAN, panoramic radiograph.

### 3.1 Horizontal tube shift technique (HTST)

**Figure 1:**
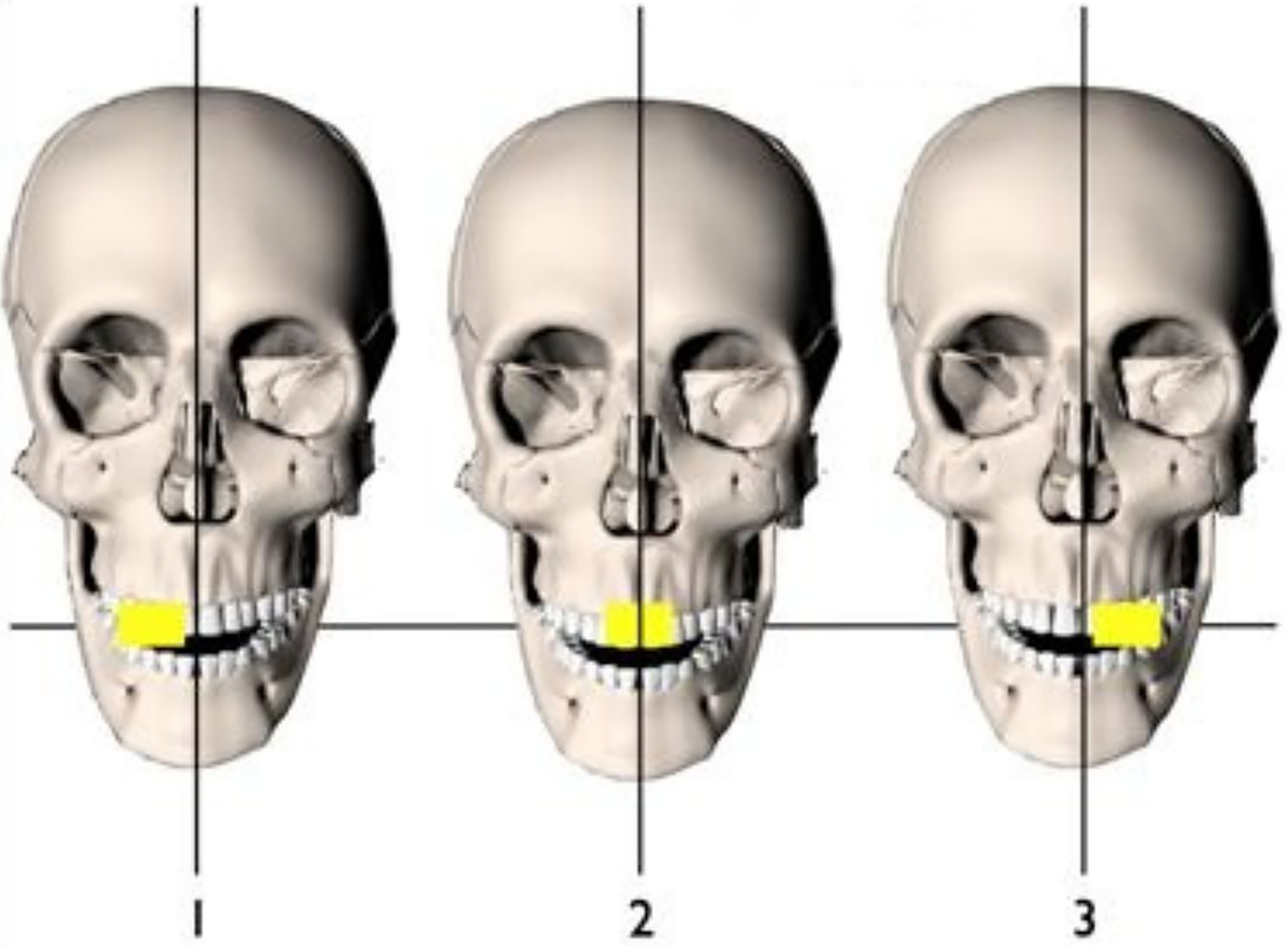
Schematic illustration of Clark's technique using horizontal tube shift.

Classically, this technique requires three periapical radiographs, one on the tooth of interest followed by one mesial and another distal to the first radiograph (13) see Figure 1. However, over the years, there has been a reduction in the number of films used for this technique, so presently only two periapical films are routinely used. While maintaining the same horizontal plane, a tube shift of 20° to 30° is made between each film. This technique is commonly referred to as Clark's technique and employs the principle of parallax to delineate the spatial relationships of an object.

### 3.2 Vertex occlusal

**Figure 2:**
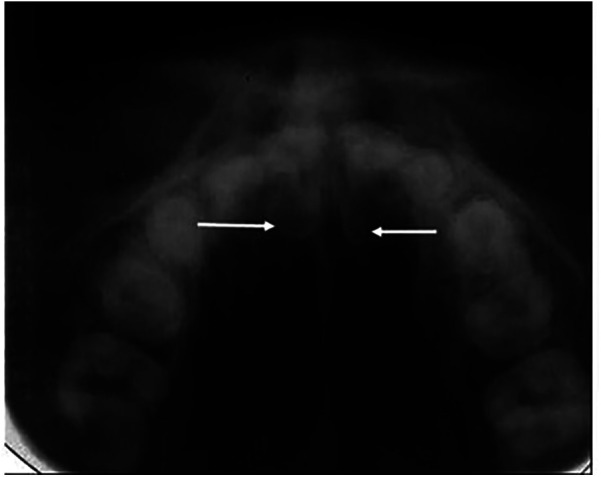
Two palatally located supernumerary teeth [arrows] on a vertex occlusal film, note the lack of clarity of images.

Localization of an impacted tooth by radiographic means is dependent on the presence of fixed points apparent both visibly and radiographically (15). To obtain the accurate location of an impacted tooth, the central ray of the x-ray beam must be directed along the long axis of those teeth in the dental arch, which is to be used as reference points. An intraoral intensifying screen may be used to reduce the radiation dose. This technique is not recommended when the voltage of the dental x-ray set is less than 65 kV (43), and it is not acceptable when a long exposure time is needed. These results in a high patient dose and a film of low diagnostic quality because of fogging from scattered radiation see Figure 2. An alternative technique was proposed for the benefit of patients and clinicians, this involved an erect Potter Bucky diaphragm or a fine stationary radiographic grid that can be employed along with the occlusal film (35). However, this technique is not recommended for Class II division 2 malocclusion patients (44), where the retroclination of the maxillary incisors results in the frontal bone obscuring the incisor region. However, probably because of the dosage issues and the quality of the poor image, this method is no longer favored.

### 3.3 Vertical tube shift technique (VTST)

**Figure 3:**
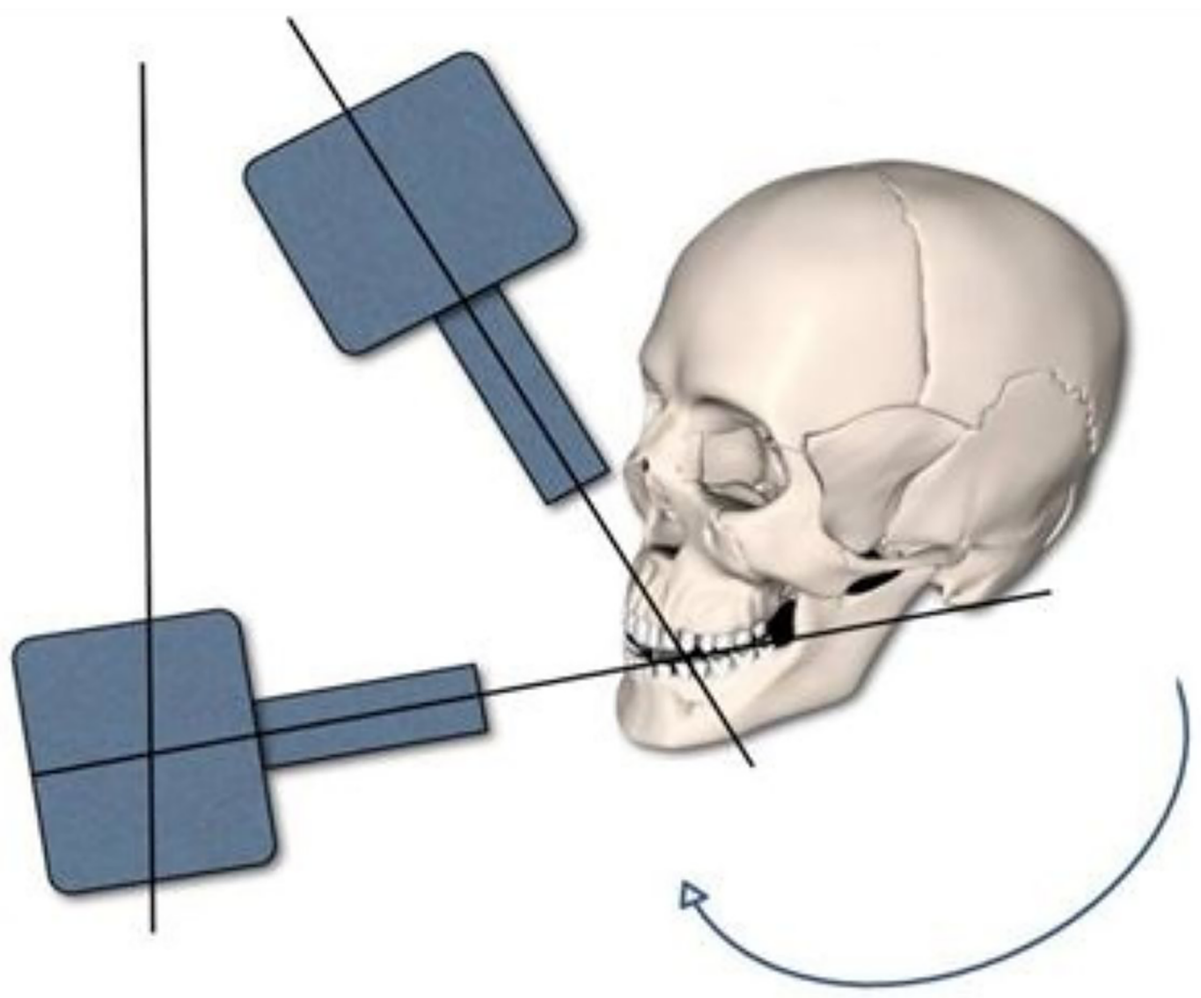
Schematic diagram of the vertical tube shift technique.

This method was introduced with the combination of panoramic and occlusal radiographs to localize an unerupted tooth in the anterior region of the maxilla (16). To obtain the panoramic radiography, the tube is positioned behind the patients’ head at an angle of −7° to the occlusal plane, and the film is placed in front of the head. Nevertheless, to aid the interpretation of tube shift, the tube can be considered to be in front of the patients' head at an effective angle of +7°, and the anterior occlusal radiograph is taken at an angle of +60° to 65° to the occlusal plane (Figure 3). Although a VTST using the PR and OR is usually not as easy to interpret, the PR-OR combination is traditionally preferred. This is because the PR, which contains information about all the teeth in both arches as well as about the jaws and surrounding structures, is often already available; it is usually taken as an initial radiograph, so only one additional exposure is required (anterior occlusal). Eventually, modifications were made in the angulations of VTST; the difference in the positioning of the tube for illustrations for occlusal radiographs 60^0^ and 70^0^ has been demonstrated. However, the recommendation is to increase the tube angle from 60^0^ to 65^0^ and 70^0^ to 75^0^ (11, 39). In a panoramic radiograph, the relationship between the images of the un-erupted ST with the reference objects is unaltered if the x-ray tube is considered to be on the facial side of the arches rather than on the lingual or buccal. The larger the distance between the impacted tooth and the image of an impacted tooth with a given x-ray tube movement, results in the easier determination of its position. Both the positions of the crown and of the root apex should be checked to gain a full picture of the position of the impacted tooth. Furthermore, it has been stated that this combination of radiographs should provide the clinician with a good diagnostic yield for the radiation dose given (44).

### 3.4 Cone beam computerized tomography [CBCT]

**Figure 4:**
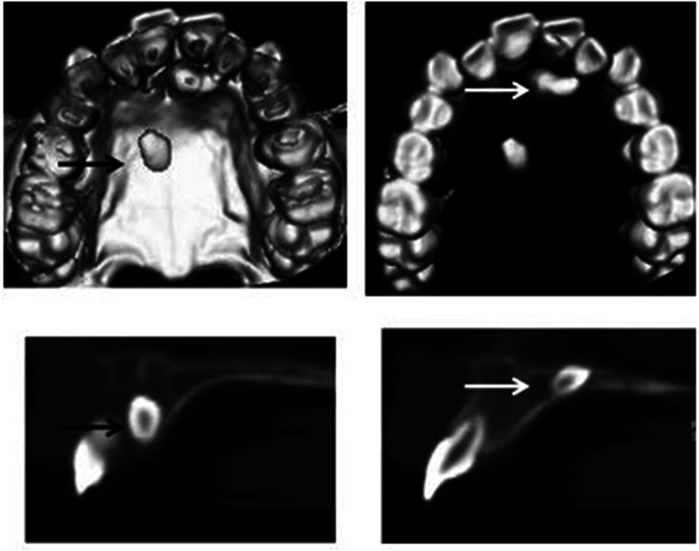
Cone beam computerized tomography 3D images are used for the localization of supernumerary teeth [arrows].

The CBCT respectively offers 3D- imaging of the maxillofacial region, providing the opportunity to study objectives in all standard plans with 3D reconstruction in multi-section views (17). The exact localization of supernumerary teeth is often difficult to assume by using conventional radiological techniques like PVTSAN or intra-oral dental films. A preoperative radiological investigation using CBCT on patients who are prepared to undergo surgery for impacted and supernumerary teeth in the frontal maxilla can more certainly indicate the nature of the pathology and hence enhance the surgical safety (Figure 4).

## 4 Discussion

A combination of clinical and radiological assessments is necessary for the diagnosis of supernumerary teeth. Most often, clinicians will take radiographs based on clinical information to confirm the diagnosis of supernumerary teeth. The localization of supernumerary teeth from radiographs is an important diagnostic adjunct to clinical assessment, diagnosis, and treatment planning. This is most important when surgical intervention is required. To evaluate the position and orientation of supernumerary teeth, the most commonly obtained radiographs are periapical, occlusal, panoramic, and lateral cephalograms. By localizing the supernumerary teeth, the site and extension of the flap margins and the amount of bone removal can be planned prior to surgical intervention. Although periapical, occlusal, and panoramic radiographs are usually able to provide the required information, these modalities do not always provide sufficient information concerning the 3-dimensional [3D] relationship of the supernumerary teeth and the surrounding structures for surgical planning (45, 46). One of the limitations of a single radiograph is its relative inability to demonstrate the relationship between two objects that are either side by side or superimposed (47). It is difficult to determine whether both are in the middle of the bone or buccal or lingual to each other. Furthermore, numerous alternative localization strategies have been articulated as a principle for interpreting buccal and lingual relationships in serial images produced from different techniques. However, different imaging modalities, ranging from intra-oral and extra-oral radiographs to computed tomography (CT) have been used for the evaluation of supernumerary teeth. The most frequently used localization techniques are the horizontal (13) and vertical tube shift (16) techniques using conventional radiographs. Stereoscopic methods (18) were used to localize the foreign bodies, such as bullets and impacted teeth prior to Clark's horizontal tube shift technique (13). Subsequently, based on this principle, various tube shift techniques have been proposed in the literature. However, the interpretation of both techniques enables the clinician to determine the relative position of the displaced tooth (48). The clinical signs provide preliminary information that later is confirmed by radiographic examination.

The treatment decisions have traditionally been based on planar 2-dimensional radiographs such as intraoral and extraoral radiographs (49). Although many localization techniques have been proposed using different combinations of radiographs, they just provide a 2-dimensional view of 3-dimensional structures. The introduction of cone-beam computerized tomography [CBCT] in dentomaxillofacial radiology has created new diagnostic possibilities, which includes evaluating supernumerary teeth (50). However, currently, CBCT has limited usage due to its high cost, low vertical resolution, and high radiation dosage (51). CBCT can be used to provide a 3-dimensional visualization of the oral maxillofacial complex, which aids in the formation of the treatment plan (52). This new imaging technique provides a rapid 3D volumetric image, with low radiation exposure than conventional CT. Using CBCT, the clinician can view the data in axial, sagittal, and coronal sections in three dimensions. Besides, it is possible to obtain periapical, panoramic, occlusal, and lateral cephalograms from a single cone beam scan. CBCT provides a 3-dimensional view with more detailed and accurate imaging compared to conventional and digital radiographs. Nevertheless, the clinician should determine the risks and benefits of imaging for each individual. Furthermore, some significant factors need to be considered; when deciding whether to purchase a CBCT device or to refer patients to imaging centers which include cost, the time required to generate images, training, data transmission and storage, knowledge about software, and accountability for the interpretation and review of the pathology. Many published studies and case reports have accepted the use of CBCT images in oral maxillofacial surgery, dental implantology, orthodontics, and pediatric dentistry because of measurement accuracy, comparisons between 2-D and 3-D images for diagnosis and treatment planning, and the clinical use of native 3-D information. The exposure dose for CBCT devices is typically in the range between 40 and 135 μSV, and the scan time generally from 5.7 to 40 s. The effective absorbed radiation dose for a complete cone beam volume tomographic image of the maxillofacial area is within the range for a full-mouth set of periapical films (53). There is controversy over the prophylactic removal of unerupted supernumerary teeth, which do not have any apparent pathological complications. It has been suggested that early removal prevents space loss and avoids extensive orthodontic treatment in the future (52). Alternatively, studies have reported an eruption rate of approximately 80% for supernumerary teeth positioned normally (54, 55). Accurate localization of supernumerary teeth is required to make a comprehensive diagnosis, determine the appropriate surgical access, and treatment planning. For a pediatric patient, knowing the exact position of supernumerary teeth is paramount to avoiding potential complications. The risk of problems associated with the supernumerary teeth in the anterior maxillary region, early diagnosis of disturbances, and proper management are considered to be important in factors growing children (56). A recent revolution of artificial intelligence (AI) when used in dentistry has given new scope for the identification of supernumerary teeth using various AI tools (57–59). The AI technologies, especially in dental imaging, enhance accuracy and efficiency to identification, and hence management of dental anomalies (57). The incorporation of artificial intelligence in dental practices is through enhancing diagnostic accuracy and making tailored treatment strategies possible. AI systems such as Diagnocat analyze, dental images used to identify supernumerary teeth and offer a comprehensive treatment planning option that demonstrates AI's capability to improve diagnostic accuracy and efficiency in dentistry (59). AI-powered tools that use deep learning neural networks are excellent at identifying and numbering teeth on panoramic x-rays, that is necessary for identifying supernumerary teeth (60, 61).

Although, CBCT and traditional radiographs are equally effective for the initial diagnosis of pathology. CBCT provides more information on the location of pathology and the presence of root resorption, which is crucial for treatment planning (62, 63). However, there has been limited research on various conventional radiographic methods for localizing impacted teeth (59, 62–64). Most of the reports in the literature focus on the localization of impacted canines and third molars, with only a few studies reporting on the localization of supernumerary teeth (65, 66). A recent study revealed that VTST outperforms HTST in accurately locating supernumerary teeth in the anterior region of the maxillary arch (65). However, the results are not statistically significant. The CBCT is better than traditional radiography because it offers accurate and authentic anatomical information with excellent surgical predictability without distortion or artifacts. It reduces costs and surgical challenges, enabling faster surgery completion (66). Researchers have reported no significant difference in localizing dilacerations, supernumerary teeth, and impacted incisors in the anterior region of the maxilla using a periapical film instead of an anterior occlusal film (2, 40). Most recently, several studies focused on using 3D imaging for the identification of the supernumerary teeth (61, 67). Toureno et al. (67) proposed a guideline for the identification and localization of supernumerary teeth in both two and three dimensions. The guideline aimed to minimize treatment errors and enhance communication among healthcare professionals and third-party administrators. CBCT provides clear 3D images that help doctors accurately locate missing teeth and other structures in the area (66–68). This is important for planning effective treatment and surgeries. Studies have shown that CBCT is more accurate at diagnosing than 2D radiographs, with an accurate preoperative finding rate (68–70). Recently various epidemiological studies used CBCT to report the prevalence of supernumerary teeth. It was truly evident that the trends of shifting from two-dimensional imaging to CBCT can better assess the number, location, shape, and position of supernumerary teeth, providing a comprehensive evaluation that is beneficial for preventing complications (69–72). Nevertheless, in situations involving multiple supernumerary teeth or when precise positioning is crucial, CBCT remains the preferred choice (72, 73). Even though CBCT excels in numerous facets, two dimensionals radiographs certain their significance in preliminary evaluations because of their economical nature and lower radiation risk (74). The is narrative review evaluates the available localization techniques when used to locate the position of impacted teeth, particularly supernumerary teeth.

## 5 Conclusion

The most commonly used localization techniques for supernumerary teeth are horizontal tube shift, vertex occlusal, vertical tube shift, and cone-beam computerized tomography. Unfortunately, conventional radiographs are only able to provide two-dimensional views of three-dimensional structures. Nevertheless, three-dimensional imaging alone can provide precise and accurate data on impacted supernumerary teeth however, the need for this film has to be justified because of the higher radiation exposure. The paper also describes the trends in the use of other various techniques for the localization of supernumerary teeth.
